# 3D printed scaffolds loaded with BMP-2 for bone defect regeneration: a systematic review and meta-analysis

**DOI:** 10.3389/fphys.2025.1641937

**Published:** 2025-07-30

**Authors:** Lei Li, Lishi Yang, Yue Yang, Jiayi Zhu, Rongnan Shi, Qi Deng, Jianxiong Wang, Fuhua Sun

**Affiliations:** ^1^ Rehabilitation Medicine Department, The Affiliated Hospital of Southwest Medical University, Southwest Medical University, Luzhou, Sichuan, China; ^2^ Department of Oncology, The Affiliated Hospital of Southwest Medical University, Southwest Medical University, Luzhou, Sichuan, China; ^3^ Department of Rehabilitation Medicine, Southwest Medical University, Luzhou, Sichuan, China; ^4^ Rehabilitation Medicine and Engineering Key Laboratory of Luzhou, Luzhou, Sichuan, China

**Keywords:** 3D printing, BMP-2, bone regeneration, systematic review, meta-analysis

## Abstract

**Background:**

Three-dimensional (3D) printing technology and bone morphogenetic protein- 2 (BMP-2) represent promising strategies for promoting bone regeneration.

**Objectives:**

This systematic review aims to assess the effects of 3D printed scaffolds loaded with BMP-2 on bone regeneration in preclinical studies.

**Methods:**

We conducted a search in the PubMed, Web of Science, and Embase databases. Based on the inclusion and exclusion criteria, we selected and evaluated original research articles investigating on the effects of 3D printed scaffolds loaded with BMP-2 *in vivo* bone regeneration. The selected studies underwent bias risk assessment and sensitivity analysis. We then performed a random effects meta-analysis to evaluate the efficacy of BMP-2 loaded 3D printed scaffolds, with results presented as standardized mean differences (SMD) and their corresponding 95% confidence intervals. Subgroup analyses were conducted based on animal species, size of bone defects, and treatment duration. This review included 17 studies for meta-analysis.

**Results:**

BMP-2 loaded 3D printed scaffolds significantly increased both the bone volume to total volume ratio (BV/TV) [2.15 (1.14, 3.16)], the percentage of new bone volume [3.07 (1.86, 4.28)], and the percentage of new bone area [3.93 (1.51, 6.35)].

**Conclusion:**

Preclinical evidence substantiates the capacity of BMP-2- functionalized 3D-printed scaffolds to promote bone regeneration through spatially controlled osteoinductive signaling. These findings provide important insights for the future application of such scaffolds in bone regeneration or repair in subsequent research.

## 1 Introduction

Despite the natural regenerative capacity of bone tissue, 10%–20% of fracture cases manifest abnormal healing or non-union (Jagodzinski and Krettek, 2007). The structural integrity of bone relies on the tightly regulated balance between resorption and formation processes, establishing a dynamic equilibrium in bone metabolism. This equilibrium is modulated by the coordinated coupling of osteoclasts and osteoblasts (Kim et al., 2020). When this balance is disrupted, the bone tissue’s ability to regenerate is compromised (Loi et al., 2016; Su et al., 2018). Aging populations and evolving lifestyle factors have escalated the prevalence of complex bone defects, particularly in instances of extensive defects, tumor resections, and skeletal abnormalities. Concurrently, complications such as osteoporosis, diabetes, and immune disorders have intensified the challenges faced by healthcare systems.

Recent research on the prevention and treatment of bone injuries has made significant progress across multiple domains. In terms of pathological treatment, therapeutic strategies for osteosarcoma and multiple myeloma are continually being refined (Liu et al., 2025; Wu et al., 2025). In the field of early diagnosis, deep learning models offer new methods for identifying primary bone tumours (Wang et al., 2025). In functional rehabilitation, novel multimodal human-exoskeleton collaborative control technologies have notably enhanced lower limb interaction performance and movement comfort for patients with motor disabilities (Kou et al., 2024). However, in the domain of bone defect repair, autologous bone grafting remains the clinical “gold standard” for treatment (De Long et al., 2007). However, its widespread application is limited by factors such as a scarcity of donor tissue, postoperative pain, and complications at the donor site (Banwart et al., 1995). Conversely, allogeneic bone grafting encounters challenges including insufficient bone viability, donor incompatibility, and an elevated risk of disease transmission (De Long et al., 2007; Giannoudis et al., 2005). Therefore, there is an urgent need for suitable synthetic or biomimetic natural materials to replicate the structure and composition of natural bone tissue, thereby facilitating the design of scaffolds for bone regeneration or repair.

In recent years, three-dimensional (3D) printing technology has emerged as a transformative tool in the field of bone regeneration research (Zhang et al., 2019; Xu et al., 2022). This technology enables the fabrication of three-dimensionally ordered scaffolds with tunable porosity, biomechanical compatibility, and site-specific bioactivity. In addition to serving as substrates for cellular adhesion and osteogenesis, these constructs must also provide mechanical support during tissue remodeling, underscoring the criticality of scaffold mechanical properties in implantation success (Ansari et al., 2022). To enhance the osteogenic potential of 3D-printed scaffolds, incorporating osteogenic inducing factors into the scaffolds presents an effective strategy. Bone Morphogenetic Protein-2 (BMP-2) serves as a potent bone formation inducer also plays pivotal roles in angiogenesis and vascular maintenance (Mikai et al., 2020). Numerous animal and clinical studies have demonstrated the significant efficacy of BMP-2 in promoting bone formation (Krishnan et al., 2017). Su et al. employed 3D printing technology to fabricate polycaprolactone/β-tricalcium phosphate (PCL/β-TCP) scaffolds, which were utilized as carriers for the delivery of BMP-2 (Park et al., 2021). They assessed the scaffolds' efficacy in promoting mandibular bone repair in dogs. In a separate study, Zhang et al. investigated the osseointegration capabilities of implants made from porous 3D-printed Ti6Al4V loaded with recombinant BMP-2 (Zhang et al., 2020). The results demonstrated that the implants containing recombinant BMP-2 exhibited significantly superior osseointegration efficiency compared to the control group.

In the field of regenerative medicine, 3D printing technology has emerged as a cutting-edge innovation in regenerative medicine, with expanding applications in clinical translation (Goh et al., 2015). Compared to traditional methods, 3D-printed scaffolds offer distinct advantages in promoting bone regeneration, particularly in terms of space maintenance. However, their application is still in the early stages of development (Ivanovski et al., 2023). As an effective bone-inducing factor, the application of BMP-2 in bone regeneration has been explored in several systematic reviews (Li et al., 2019; Ma et al., 2022; Teng F. et al., 2019). However, there has yet to be a review specifically addressing the use of 3D-printed scaffolds loaded with BMP-2 in promoting bone regeneration. This review synthesizes preclinical evidence supporting the synergistic potential of BMP-2-functionalized 3D-printed constructs in bone tissue engineering, with emphasis on mechanistic insights, material innovations, and translational challenges.

## 2 Methods

The protocol was registered with PROSPERO under registration number CRD420250631196.

### 2.1 Search strategy

A systematic literature review was conducted using the keywords “3D print,” “bone regeneration,” “bone defect regeneration,” “bone formation,” “osteogenesis,” “bone reconstruction,” “bone repair,” “bone healing,” “bone morphogenetic protein,” and “BMP” across the PubMed, Web of Science, and EMBASE electronic databases. Each database was queried with an appropriate search strategy, with the search limited to the English language and no restrictions placed on publication dates. The final search was conducted on 31 December 2024. Additionally, the reference lists of the included studies were evaluated to incorporate any other relevant articles that were not identified through electronic searches.

### 2.2 Inclusion criteria

*In vivo* studies assessing the efficacy of BMP-2-loaded 3D-printed scaffolds in bone regeneration procedures were included;

Experimental studies were required to include research models involving the use of BMP-2-loaded 3D-printed scaffolds for the treatment of any type of bone defect;

This study places no restrictions on the types of materials for 3D-printed scaffolds (including but not limited to synthetic polymers, natural polymers, ceramics, or composites), the dosage of BMP-2 (ranging from low to high concentrations), or the loading methods (such as physical adsorption, chemical coupling, or microsphere encapsulation). Additionally, the experimental design includes animal models across various sizes, from small (e.g., rats) and medium (e.g., rabbits) to large (e.g., dogs), while maintaining an open approach to the selection of materials for scaffold synthesis.

### 2.3 Exclusion criteria

Studies involving 3D-printed scaffolds without BMP-2 loading, as well as those featuring non-3D-printed scaffolds with BMP-2, were excluded based on the title and abstract;

Articles that do not present *in vivo* studies will also be excluded;

*In vivo* studies with incomplete result reporting, such as those lacking specific values for outcome measures, were excluded from the analysis;

Articles that implemented additional interventions following the implantation of 3D-printed scaffolds were excluded;

Abstracts, reviews, letters, and scholarly articles were all excluded.

### 2.4 Selection of the studies

Two authors independently conducted an initial screening of the research reports. If an abstract was absent, the full text was obtained and examined. All articles that passed the initial screening required the acquisition of the full text for further evaluation against the inclusion and exclusion criteria. Any disagreements were resolved through direct discussion. The reasons for excluding certain articles were duly recorded.

### 2.5 Data extraction

Two authors independently extracted and analysed the data from the included articles according to a pre-designed data collection form, ensuring systematic documentation. The aim was to conduct both quantitative and qualitative assessments of the efficacy of BMP-2-loaded 3D-printed scaffolds in bone regeneration surgeries. The primary outcome measures were the percentage of new bone formation volume, the percentage of new bone formation area, and BV/TV. Additional data included species, age, weight, sex, sample size, characteristics of the bone defect models (including site and size), 3D printing technology used, porosity, average pore diameter, treatment duration, and the type of biomaterial used for the 3D-printed scaffolds.

### 2.6 Risk-of-bias analysis

This review employed the ARRIVE (Animal Research: Reporting *In Vivo* Experiments) guidelines to assess the risk of bias and other methodological standards in the evaluation of bias risk within animal studies (Tumedei et al., 2019).

Quality criteria taken into consideration were as follows:

1. Ethical statement
2. Experimental procedures
3. Experimental animals
4. Randomization (selection bias)
5. Allocation concealment (selection bias)
6. Blinding of the evaluator (detection bias)
7. Sample size calculation
8. Incomplete outcome data (attrition bias)
9. Statistical analysis appropriateness
10. Financial conflict of interest

The quality assessment of the selected articles was conducted independently by two authors, using a blinded approach for the authors’ names, institutions, and journal titles. All criteria were evaluated as “high”, “low”, or “unclear”. The studies with at least 7/10 appropriate parameters and no inappropriate parameters were considered as low. Otherwise, the studies were classified as high risk. This study used the risk of bias tool developed by the Cochrane Collaboration for bias risk assessment and conducted the analysis using RevMan 5.4.1 software. Any disagreements between the authors were resolved through discussion and consensus.

### 2.7 Statistic analysis

Data were analyzed using RevMan 5.4.1, and articles selected according to the inclusion and exclusion criteria were included in the meta-analysis. This analysis was restricted to comparable studies reporting the same outcome measures. The primary outcomes—percentage of new bone volume, percentage of new bone area, and BV/TV—were assessed using the meta-analytic method to calculate the standardised mean difference (SMD) between the experimental and control groups. The I^2^ statistic was used to assess the statistical heterogeneity between the included studies (Fu et al., 2021), a random-effects model was employed in the presence of high heterogeneity. We utilized forest plots to display effect sizes along with their confidence intervals (CIs). Subgroup analyses and investigations of heterogeneity were conducted to explore potential sources of heterogeneity based on the following factors: species of animals, size of bone defects, and treatment duration. Secondly, We systematically assessed publication bias using the following methods: first, a visual inspection was conducted via funnel plots, followed by statistical validation using Begg’s rank correlation test (Begg and Mazumdar, 1994) and Egger’s linear regression test (Egger et al., 1997). If significant publication bias was detected, the Trim and Fill method (Duval and Tweedie, 2000) was employed to correct the effect size, estimating the impact of potentially missing studies and adjusting the combined effect value accordingly. Moreover, a sensitivity analysis using a meta-based influence analysis was conducted to assess the stability of the results by eliminating the impact of a small sample size. Finally, in the meta-analysis, results were considered statistically significant when P < 0.05.

## 3 Results

### 3.1 Study selection and study characteristics

**FIGURE 1:**
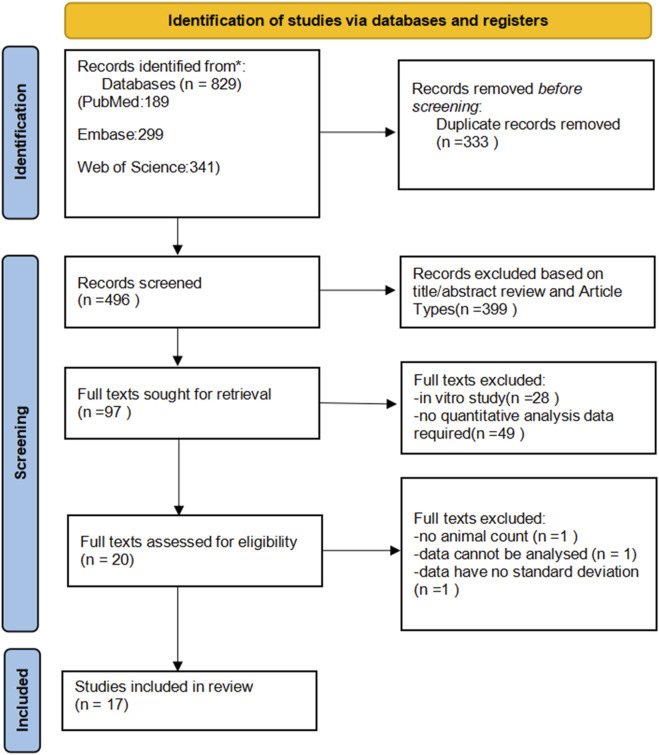
Flowchart of the study selection process.

A total of 189 papers were identified from PubMed, 341 from Web of Science, and 299 from Embase. After removing duplicates in EndNote, 496 papers remained. Following a preliminary screening based on titles and abstracts, 97 papers were selected for full-text analysis. Ultimately, after full-text screening, 20 papers were included, of which 17 studies were selected for meta-analysis (Figure 1) (Cha et al., 2021; Han et al., 2020a; Han et al., 2020b; Ishack et al., 2017; Jang et al., 2019; Khvorostina et al., 2023; Kim et al., 2018; Kolan et al., 2020; Kou et al., 2021; Lee et al., 2020; Ma et al., 2023; Maia-Pinto et al., 2020; Shim et al., 2014a; Shim et al., 2014b; Teng F. Y. et al., 2019; Wang et al., 2021; Zhuang et al., 2022), Three additional studies were excluded from the meta-analysis due to the lack of standard deviation, a control group data value of zero, and insufficient sample size information (Lee et al., 2023; Poldervaart et al., 2013; Zhang et al., 2022).

The studies utilized five different animal species, with the most commonly employed being rats (9 studies), followed by rabbits (6 studies), dogs (1 study), and mice (1 study). The majority of these studies used male animals (10 studies), while a minority used female animals (1 study), and 6 studies did not specify the sex of the animals. Various animal models of bone defects were investigated, including cranial defects (12 studies), tibial defects (1 study), radial defects (1 study), mandibular defects (1 study), femoral distal bone defects (1 study), and mastoid occlusion (1 study). Seven studies reported the BMP-2 dosages loaded onto the scaffolds. However, the dosages varied significantly, with the minimum loading being 0.3 µg and the maximum at 100 µg. Notably, 10 studies did not disclose the BMP-2 loading amounts for their respective scaffolds. Table 1 also summarises key information from each study, including the animal species and models used, 3D printing scaffold materials and techniques, scaffold structural characteristics (such as porosity and pore size), BMP-2 dosage, follow-up period, and outcome measures. Nine studies conducted micro-CT assessments of the retrieved samples, six studies reported histomorphometry analyses, one study performed microcomputed tomography analysis, and one study utilized cross-sectional SEM to analyze the amount of bone regeneration.

**TABLE 1:** BMP-2-loaded 3D printed scaffolds used in each study.

Author/ year	Species	Age	Weight	Sex	N	Animal model	Template materials	Porosity	Average pore size	Printing technique	BMP-2 dose	Defect diameter	Follow-up period	Evaluation method	Outcome measures
Jang et al. (2019)	SD rats	Unclear	250∼300 g	Male	20	Mastoid obliteration model	PCL	Unclear	300-400um	Three-axis printing system (DTR32210 T-SG, DASA Robot, Seoul, South Korea)	Ⅰ group: 10 µg Ⅲ group: 5 µg	N	12 W	Histologic, histomorphometric	New bone areas
Khvorostina et al. (2023)	Wistar rats	Unclear	200 g	Unclear	9	Skull defects	Sodium alginate (SA)	Unclear	Unclear	3D cryoprinting	Unclear	7.5 mm	8 W	micro-CT	BV/TV, new bone volume
Ku et al. (2021)	SD rats	Unclear	Unclear	Male	24	Skull defects	PCL	50%	524.34 ± 9.62 µm	The Kagome-structure scaffold deposited by 3D printing	1 µg per scaffoerd	2 mm	2 W; 4 W; 8 W	micro-CT	New bone volume
Kim et al. (2018)	New Zealand white rabbits	>3 months	2∼3 kg	Male	4	Skull defects	Hydroxyapatite (HAp), water-based binder	64.96% ± 0.87%	Unclear	3D CAD program (SolidWorks, Concord, MA, United States of America), 3DP equipment (Z350, Z-Corporation)	Unclear	6 mm	8 W	micro-CT	New bone volume
Wang et al. (2021)	New Zealand rabbits	Unclear	Unclear	Female	46	Distal femur cylindrical bone defect	Ti6Al4V	70.13% ± 2.43%	791.97 μm	Electron beam melting (EBM) machine (Q10, Arcam, Gothenburg, Sweden)	Unclear	6 mm	3 month	micro-CT	BV/TV
Han et al. (2020a)	SD rats	8 W	240∼260 g	Male	34	Skull defects	Calcium deficient hydroxyapatite (CDHA), collagen, α-TCP	CDHA: 87.20% ± 0.68% BMP: 86.55% ± 0.77%	CDHA: 532.5 ± 37.6 μm BMP: 524.0 ± 25.5 μm	Low temperature printing	Unclear	8 mm	8 W	micro-CT	BV/TV; New bone areas
Ma et al. (2023)	SD rats	Unclear	180∼220 g	Male	12	Skull defects	Poly-(lactide-co-glycolide)/ mesoporous bioactive glass (PLGA/MBG), Zeolitic imidazolate framework-8 (ZIF-8)	Unclear	Unclear	3D bioprinter (Envision TEC, Germany)	100 µg per scafford	5 mm	12 W	Histologic, histomorphometric	New bone areas
Lee et al. (2020)	Beagles	Unclear	Unclear	Unclear	8	Mandible bone defects	PCL, β-TCP	70%–75%	External layer: >300 μm Internal layer: >600 μm	3D modeling software (3-Matic Research 9.0, Materialise, Leuven, Belgium)	Unclear	Non-circular	12 W	Histologic, histomorphometric	New bone areas
Shim et al. (2014a)	New Zealand white rabbits	12 W	3.3∼3.5 kg	Unclear	36	Skull defects	PCL, PLGA, β-TCP	50%	250 µm	Multi-head deposition system (MHDS; a type of 3D printing system)	Unclear	8 mm	4 W; 8 W	Histologic, histomorphometric	New bone volume
Teng et al. (2019a)	New Zealand White rabbits	Unclear	3.5∼4.5 kg	Unclear	18	Skull defects	Ti_6_Al_4_V, Ca, P	Unclear	600 μm	3D printing (EOSINT M280, Germany)	Unclear	20 mm	6 W; 12 W	Cross-sectional SEM	New bone volume
Ishack et al. (2017)	C57Bl/6 and adenosine A2A knockout (KO) mice	Unclear	Unclear	Unclear	45	Skull defects	Hydroxyapatite (HA)/Beta-Tri-Calcium Phosphate (β-TCP)	Unclear	250 μm	3-D direct-write microprinter gantry robot system to extrude the colloidal ink (Aerotech Inc. Pittsburgh, PA)	Unclear	3 mm	2 W; 4 W; 8 W	micro-CT	New bone volume
Han et al. (2020b)	New Zealand White Rabbits	4 months	2.5∼2.99 kg	Male	21	Tibia defect	PLA, gelatin and alginate	58.86%	0.56930 µm	3D printer mounting box (Replacator™2, Makerbot, New York City, United States)	0.3 µg per scafford	10 mm	4 W	micro-CT	BV/TV
Maia-Pinto et al. (2020)	Wistar rats	Unclear	300∼400 g	Unclear	45	Skull defects	PLA, apatite	49.09% ± 3.22%	Unclear	3D Cloner FDM machine (Microbrás, Toledo, Paraná, Brazil)	5 µg per scafford	8 mm	3 months; 6 months	Histomorphometric	New bone volume
Zhuang et al. (2022)	SD rats	Unclear	200∼250 g	Male	40	Skull defects	PCL	Unclear	200–300 μm	3D Bioplotter (Bio-x, Cellink, Sweden)	1 µg per scafford	8 mm	12 W	micro-CT	BV/TV
Kolan et al. (2020)	SD rats	12 W	∼350 g	Male	12	Skull defects	Borate glass	Cubic: 54% ± 1% Diamond: 47% ± 1%	1.0 ± 0.04 µm	Selective laser sintering (SLS) process	1 µg per scafford	4.6 mm	6 W	Histologic, histomorphometric	New bone areas

### 3.2 Risk of bias

**FIGURE 2:**
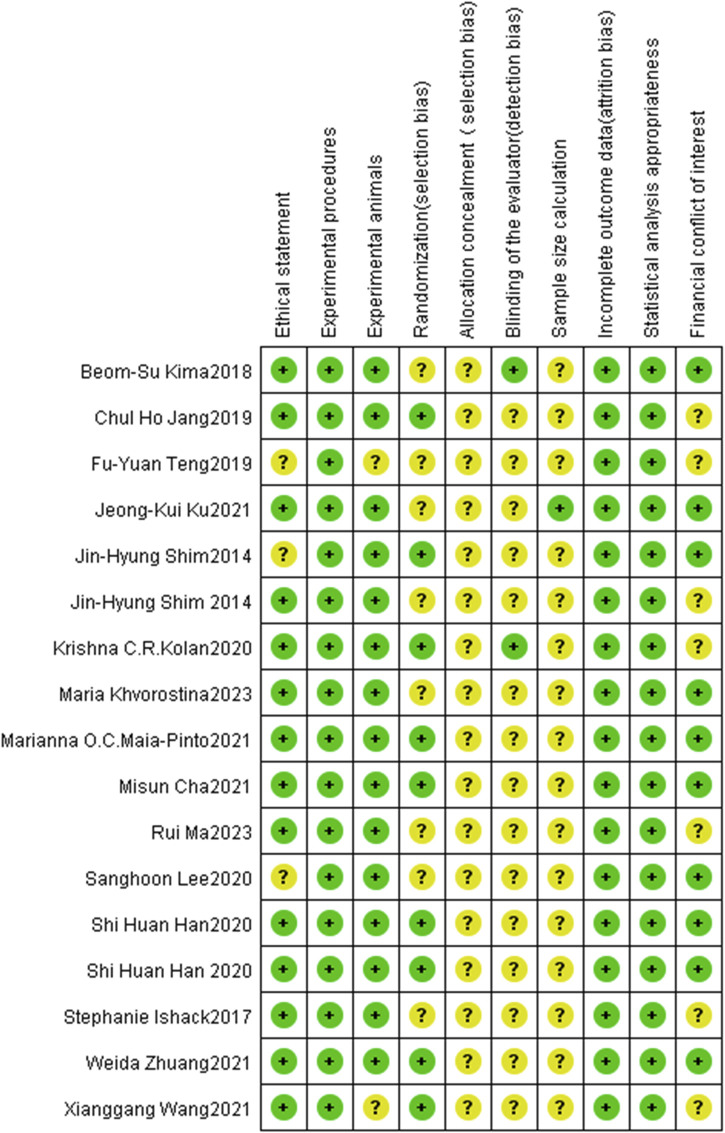
Risk-of-bias graph: review authors’ judgments about each risk-of-bias item presented as percentages across all included studies. + = low risk (the criteria was met, green circle); ? = unclear risk if the criteria was met or not (yellow circle).

**FIGURE 3:**
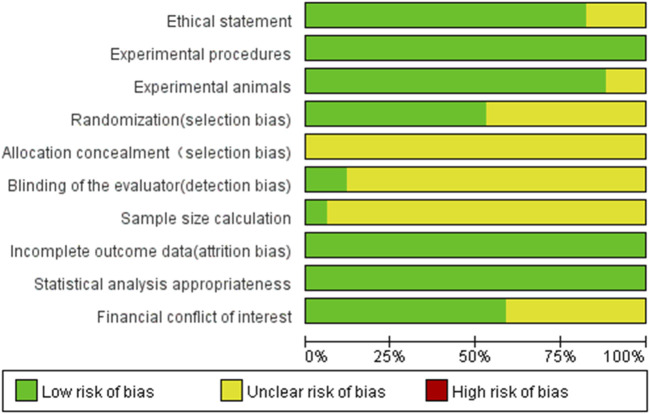
Risk-of-bias summary: review authors’ judgments about each risk-of-bias item for each included study. The colors have the same meaning as in Figure 2.

Figure 2 illustrates the risk of bias ratings for all included articles. Based on the assessment criteria, only eight studies (Cha et al., 2021; Han et al., 2020a; Han et al., 2020b; Kim et al., 2018; Kolan et al., 2020; Ku et al., 2021; Maia-Pinto et al., 2020; Zhuang et al., 2022) were classified as having a low risk of bias, while all other included studies exhibited a high risk of bias. As shown in Figure 3, all articles described the experimental procedures; however, none provided details on the methods for concealing allocation sequences. Only 5.9% of the studies included calculations for the required sample size, while 82.4% mentioned an ethical statement. Furthermore, 88.2% of the studies provided relevant information about the experimental animals, including sex, age, and sample size. Random housing of animals during the experimental process was reported in 52.9% of the studies. In the final analyses of the results, only 11.8% reported blinding of assessors across different groups. Despite this, all studies employed appropriate statistical methods and reported no incomplete outcome data. Lastly, 58.8% of the studies indicated that there were no conflicts of economic interest. In conclusion, the risk of selection and detection bias remains unclear in a small number of studies, while the majority are classified as low-risk for bias.

### 3.3 Meta-analysis

This meta-analysis included a total of 17 studies and focused on three primary outcomes: BV/TV, new bone volume, and new bone area.

#### 3.3.1 BV/TV

**FIGURE 4:**
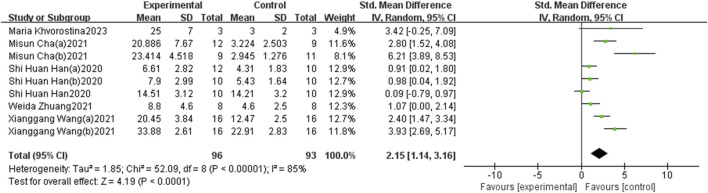
The forest plot: the effects of 3D-printed scaffolds loaded with BMP-2 on BV/TV, compared with controls.

The six identified references, comprising nine sets of research data, evaluated BV/TV across 189 3D-printed scaffolds (96 in the experimental group and 93 in the control group). Of these, two studies reported no significant differences, while seven indicated a positive effect of the experimental group compared to the control group. Overall, BMP-2-loaded 3D-printed scaffolds demonstrated significant statistical benefits for BV/TV, with a pooled estimate of the standardised mean difference (SMD) and its 95% confidence intervals (SMD = 2.15, 95% CI = 1.14–3.16). However, the heterogeneity test revealed an I^2^ value of 85%, indicating a high degree of heterogeneity (Figure 4). The sensitivity analysis, conducted by omitting individual studies, revealed no significant alterations in the pooled effect size, indicating that the results were robust and reliable (Figure 7A).

Subgroup analyses were conducted based on different animal species, sizes of bone defects, and treatment durations. All analyses indicated a positive impact of BMP-2-loaded 3D-printed scaffolds on bone regeneration (Table 2). The results suggest that the diameter of the bone defects may be a primary contributor to the observed high heterogeneity.

**TABLE 2:** BV/TV: stratified analysis of 3D printed scaffolds loaded with BMP-2 vs. Control.

Variable	Subgroup	N	Effect estimate	I^2^	P
Species of animals	Rat	83	2.44 (0.63, 4.24)	87%	P = 0.008
	Rabbit	106	2.01 (0.74, 3.27)	85%	P = 0.002
Bone defect diameter	≥8 mm	119	1.69 (0.58, 2.81)	84%	P = 0.003
	<8 mm	70	3.11 (1.93, 4.29)	47%	P < 0.0001
Treatment duration	>8 W	80	2.44 (0.94, 3.94)	83%	P = 0.001
	≤8 W	109	2.01 (0.69, 3.32)	84%	P = 0.003

#### 3.3.2 New bone volume

**FIGURE 5:**
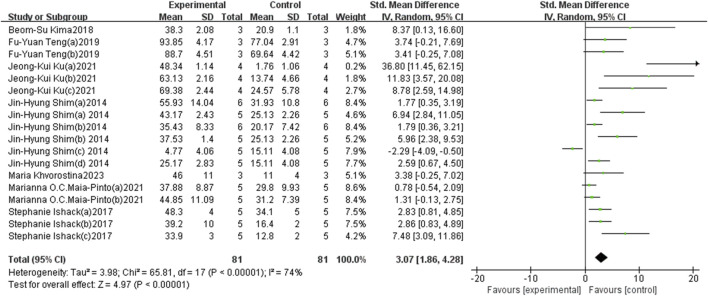
The forest plot: the effects of 3D-printed scaffolds loaded with BMP-2 on new bone volume, compared with controls.

A total of eight identified references and 18 sets of research data were analyzed, encompassing 162 3D-printed scaffolds (81 in the experimental group and 81 in the control group) to evaluate the impact of BMP-2-loaded 3D-printed scaffolds on new bone volume. Five studies reported no significant differences. Compared to their corresponding control groups, only one study reported a negative effect in the experimental group, while 12 studies demonstrated positive effects on the experimental group. In brief, the beneficial effect of BMP-2-loaded 3D-printed scaffolds on new bone regeneration was statistically significant, as indicated by the overall effect size of the standardised mean difference (SMD) and its 95% confidence intervals (SMD = 3.07, 95% CI = 1.86–4.28). However, the heterogeneity test revealed a high level of heterogeneity (I^2^ = 72%) (Figure 5). After excluding individual trials in the sensitivity analysis, the pooled effect size remained largely unchanged, indicating that the results were robust and trustworthy (Figure 7B).

Subgroup analyses were further conducted based on different animal species, sizes of bone defects, and treatment durations. All analyses revealed a positive effect of BMP-2-loaded 3D-printed scaffolds on bone regeneration (Table 3). The analysis results indicate that animal species and treatment duration may be the primary sources of heterogeneity. Due to the use of non-circular defect models in four of the research data included in the bone defect model, these studies were excluded from the subgroup analysis.

**TABLE 3:** NBV: stratified analysis of 3D printed scaffolds loaded with BMP-2 vs. Control.

Variable	Subgroup	N	Effect estimate	I^2^	P
Species of animals	Mice	30	3.61 (1.57, 5.66)	48%	P = 0.0005
	Rat	50	3.70 (0.99, 6.42)	76%	P = 0.007
	Rabbit	82	2.76 (0.99, 4.53)	78%	P = 0.002
Bone defect diameter	≥8 mm	56	1.52 (0.84, 2.20)	0%	P < 0.0001
	<8 mm	66	5.47 (3.00, 7.95)	61%	P < 0.0001
Treatment duration	>8 W	26	1.17 (0.23, 2.12)	0%	P = 0.02
	≤8 W	136	3.68 (2.13, 5.23)	77%	P < 0.0001

#### 3.3.3 New bone areas

**FIGURE 6:**
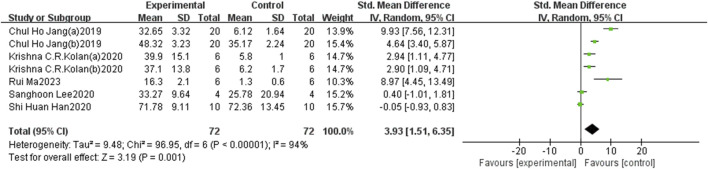
The forest plot: the effects of 3D-printed scaffolds loaded with BMP-2 on new bone areas, compared with controls.

**FIGURE 7:**
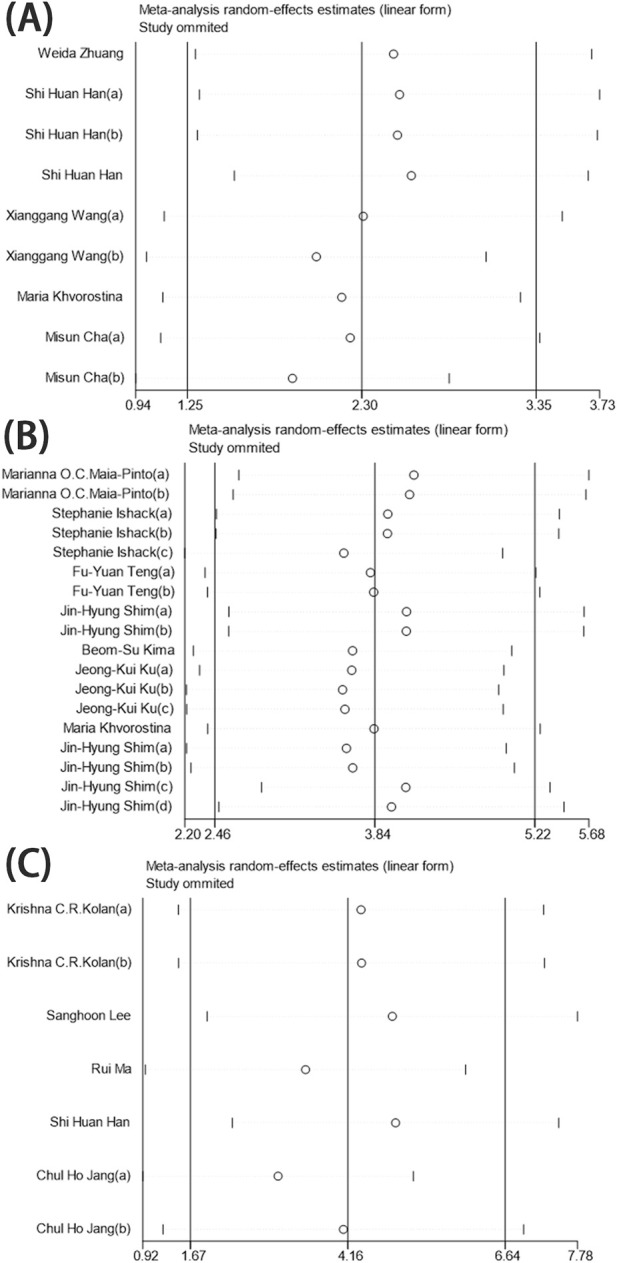
Sensitivity analysis. **(A)** BV/TV. **(B)** New bone volume. **(C)** New bone areas.

The five identified references comprising seven research data, involving 144 3D-printed scaffolds (72 experimental and 72 control groups), were evaluated to assess the percentage of new bone areas. Two studies found no statistically significant impact on new bone formation, while the remaining five reported positive effects. Overall analysis indicated that BMP-2-loaded 3D-printed scaffolds promote new bone formation, with an overall effect size of the standardised mean difference (SMD) and its 95% confidence intervals (SMD = 3.93, 95% CI = 1.51–6.35). Heterogeneity testing showed a high level of heterogeneity (I^2^ = 94%) (Figure 6). The results did not show significant variations in the pooled effect size following the exclusion of individual trials during the sensitivity analysis, implying that the findings were consistent and reliable (Figure 7C).

### 3.4 Publication bias analysis

Our publication bias analysis revealed the following: visual inspection of the funnel plots showed that only New bone areas did not exhibit significant asymmetry (Supplementary Figure S1C), whereas both BV/TV (Supplementary Figure S1A) and New bone volume (Supplementary Figure S1B) displayed significant asymmetry. Statistical tests further confirmed these findings: Begg’s test (P = 0.133) and Egger’s test (P = 0.056) for New bone areas did not reach significance, while BV/TV (Begg P = 0.029, Egger P = 0.037) and New bone volume (Begg and Egger P < 0.001 for both) demonstrated significant publication bias (Supplementary Table S1). After correction using the Trim and Fill method, the combined effect size for BV/TV increased from 2.15 (95% CI: 1.14–3.16, P < 0.001) to 5.534 (1.843–16.618, P = 0.002), and for New bone volume, it increased from 3.07 (1.86–4.28, P < 0.001) to 6.308 (1.486–26.767, P = 0.013) (Supplementary Table S2).

## 4 Discussion

A systematic analysis of 17 published animal studies investigating the effects of BMP-2-loaded 3D-printed scaffolds on bone regeneration was conducted. The following is a brief summary of these research findings: (1) BMP-2-loaded 3D-printed scaffolds can promote bone regeneration; (2) these scaffolds tend to show more pronounced therapeutic effects in cases of smaller bone defect areas; (3) while a longer duration of treatment is generally expected to enhance bone regeneration, the subgroup analysis of new bone volume revealed contrary results, potentially due to an insufficient number of studies. We believe that, with more research, a clearer correlation may be observed between longer treatment durations and improved bone regeneration outcomes. The meta-analysis substantiates the therapeutic potential of BMP-2-loaded 3D-printed scaffolds in bone regeneration, thereby substantiating their feasibility for clinical translation.

We assessed statistical heterogeneity using the I^2^ statistic (I^2^>50% indicating the use of a random-effects model) (Bai et al., 2018). The results showed that even with the random-effects model, there was still considerable heterogeneity for BV/TV (I^2^ = 85%), new bone volume (I^2^ = 72%), and new bone areas (I^2^ = 94%). Subgroup analyses revealed significant factors influencing bone regeneration outcomes. For the BV/TV measure, the diameter of the bone defect was the primary source of heterogeneity (<8 mm: I^2^ = 47%, ≥8 mm: I^2^ = 84%). For new bone volume, animal species (mice, rats, and rabbits had I^2^ values of 48%, 76%, and 78%, respectively), bone defect diameter (<8 mm: I^2^ = 61%, ≥8 mm: I^2^ = 0%), and treatment duration (≤8 weeks: I^2^ = 77%, >8 weeks: I^2^ = 0%) significantly impacted heterogeneity. Due to insufficient data, no further subgroup analysis was conducted for new bone areas.

Notably, regarding species differences, the mouse model (I^2^ = 48%) demonstrated the best experimental consistency, suggesting that smaller animal models may be more suitable for standardised bone regeneration studies. When the bone defect diameter was ≥8 mm, new bone volume showed extremely low heterogeneity (I^2^ = 0%), significantly outperforming smaller defects (I^2^ = 61%), likely because larger defects provide a more stable environment for evaluating bone regeneration, as their repair process is more dependent on the osteogenic effects of the scaffold and BMP-2 rather than local self-healing capacity. Additionally, a treatment duration exceeding 8 weeks also eliminated heterogeneity in new bone volume (I^2^ = 0%), while short-term treatment (≤8 weeks) maintained higher heterogeneity (I^2^ = 77%), likely because the critical phase of bone repair is typically completed within 8 weeks. Future research should consider a treatment period of ≥8 weeks. These findings provide important insights for optimising bone regeneration experimental design: prioritising the use of mouse models, employing standardised defect sizes ≥8 mm, and ensuring a minimum treatment observation period of 8 weeks will significantly enhance the comparability and reproducibility of research outcomes.

For the assessment of publication bias, despite significant bias being observed in the funnel plot visual inspection, Begg’s rank correlation test, and Egger’s linear regression test for BV/TV and new bone volume, the effect size direction did not reverse after correction using the Trim and Fill method, confirming the robustness of the results. Notably, the increase in effect size after correction suggests that the original analysis may have underestimated the true effect, but the significantly widened confidence intervals indicate increased uncertainty, which may be due to the additional variability introduced by the imputation of potentially missing studies. Furthermore, sensitivity analysis further confirmed the reliability of the original combined effect size, indicating that the conclusions of the meta-analysis were not influenced by potential bias or heterogeneity from individual studies, thereby enhancing the credibility of the research findings.

In bone regeneration, 3D printing has emerged as a transformative technology, enabling precise spatial control of scaffold architecture to conform to complex anatomical defect geometries while facilitating controlled therapeutic agent release to maximize efficacy (van der Heide et al., 2022). Furthermore, the customized artificial scaffolds produced not only significantly enhance the accuracy and safety of surgical procedures but also expedite the recovery of patients with bone injuries. Currently, 3D-printed metallic devices, such as those made from titanium, are widely used for bone fixation and craniofacial defect reconstruction. However, their lack of porosity limits their ability to promote bone repair or regeneration. Preformed titanium or metallic scaffolds have the potential to transform existing surgical methods for bone defect reconstruction. A key challenge lies in ensuring that these scaffolds not only provide adequate structural support to prevent soft tissue collapse, facilitate cellular residence, and possess bone inductive properties, but also promote the transmission of mechanical forces between skeletal components (Ivanovski et al., 2023). The porosity of scaffolds is crucial for cellular growth and proliferation; however, increased porosity often results in decreased mechanical performance. Therefore, careful design is essential to ensure sufficient mechanical strength while maintaining an appropriate level of porosity (Karageorgiou and Kaplan, 2005). Secondly, ceramics have been explored, with a published study investigating the use of 3D-printed ceramics for alveolar bone regeneration (Mangano et al., 2021). Seven years post-implantation, regenerated bone specimens were collected and analyzed. Histological evaluations demonstrated that the new bone was in close contact with the scaffold, and over time, the biomaterial was progressively resorbed while the proportion of newly formed bone gradually increased. Lastly, 3D-printed polymers, such as hydrogels, polycaprolactone, and polylactic acid, have been investigated. A case report by Schuckert et al. demonstrated the successful application of 3D-printed polymers in a patient with anterior mandible defects caused by peri-implant bone loss, promoting new bone formation (Schuckert et al., 2009). Within 6 months, imaging and subsequent histological analyses revealed substantial bone regeneration, enabling the successful placement of dental implants into the regenerated bone tissue.

Although 3D-printed scaffolds are fabricated from highly biocompatible materials, they may still encounter several challenges upon implantation in patients. Like all implantable biomaterials, 3D-printed scaffolds can elicit foreign body reactions (FBRs). When the scaffold is implanted in a bone defect area, proteins in the blood, such as vitronectin and fibrinogen, adhere to its surface, subsequently recruiting and activating immune cells (Liu et al., 2021). Immune cells such as macrophages and lymphocytes release inflammatory mediators that trigger an acute inflammatory response. A proper immune response is essential for initiating bone regeneration (Bahney et al., 2019), while an inappropriate immune response may lead to chronic inflammation. If the inflammatory stimulus persists, acute inflammation can evolve into chronic inflammation, resulting in the formation of fibrous capsules that further exacerbate tissue damage and hinder repair processes (Anderson et al., 2008).

Macrophages have garnered significant attention due to their diverse functions and remarkable plasticity. Generally, macrophages can induce the osteogenic differentiation of mesenchymal stem cells through paracrine factors and exosomes. The regulatory molecules secreted by macrophages, including inflammatory cytokines, BMP-2, transforming growth factor-beta (TGF-β), and vascular endothelial growth factor (VEGF), play crucial roles in the osteogenic process. Following the implantation of 3D-printed scaffolds, macrophages serve as the first line of host immunity, polarizing into two distinct phenotypes, M1 and M2, in response to microenvironmental signals. M1 macrophages typically infiltrate the site of injury caused by the implant and are responsible for debris clearance. Research has indicated that M1 macrophages adhered to the surface of biomaterials can sustain and even enhance the secretion of pro-inflammatory cytokines such as interleukin-1β (IL-1β), tumor necrosis factor-alpha (TNF-α), and interleukin-6 (IL-6) without undergoing a phenotypic switch (Jones et al., 2007), Given that 3D-printed scaffolds are intended to remain within the body for an extended duration, the persistence of M1 macrophages may lead to chronic inflammatory responses, foreign body reactions (FBRs), and fibrous encapsulation, ultimately resulting in the failure of biomaterial implantation. However, biomaterials with immunoregulatory properties can prompt macrophages to mount an appropriate immune response, releasing cytokines that facilitate the osteogenic differentiation of mesenchymal stem cells, ultimately promoting the formation of new bone (Han et al., 2017; Li et al., 2021; Mallapragada Narasimhan, 2008). Therefore, creating a microenvironment that integrates immunomodulatory and osteogenic functions is crucial for the regeneration of 3D bone tissue.

In addition to the critical structural design and immunomodulatory functions of the scaffold, it must also possess osteoinductive properties. This can be achieved by incorporating mineralized materials, such as hydroxyapatite (HA) or tricalcium phosphate (TCP), as well as adding bioactive molecules that promote angiogenesis and bone regeneration, such as BMP-2, TGF-β, or other growth factors. Our findings demonstrate that 3D-printed scaffolds loaded with BMP-2 exhibit significantly enhanced regenerative effects in new bone formation compared to the control group.

BMP-2 is one of the most important endogenous growth factors and plays a crucial role in the process of bone regeneration. It regulates cell differentiation, thereby critically influencing bone formation (Bragdon et al., 2011; Rosen, 2009). Despite the significant efficacy of BMP-2 in osteoinduction, an increasing body of research has reported complications associated with its clinical application, such as failures in bone regeneration and bone resorption (James et al., 2016). In environments with scarce bone marrow, such as tooth extraction sites and the calf, BMP-2 can effectively induce bone formation. However, in areas rich in bone marrow, such as the mandible and femoral marrow, the transplantation of BMP-2 may lead to bone resorption (Nguyen et al., 2019). Furthermore, while higher doses of BMP-2 may be necessary for the recruitment and differentiation of mineralisation-bridging progenitor cells (Seeherman, 2001), excessive use of BMP-2 can precipitate a range of complications, including bone dissolution, haematoma, and particularly ectopic bone formation due to the overgrowth of osteoblasts (Tannoury and An, 2014). Therefore, the development of a controlled BMP-2 delivery system is of paramount importance (Boerckel et al., 2011).

To date, various types of biomaterial carriers have been designed and developed, which can generally be categorised into two broad classes: (i) chemically fixed carriers and (ii) physically encapsulated carriers (Lee et al., 2011). Current synthetic bone materials, such as bioactive glass, calcium phosphate cements, polymethyl methacrylate, and composites, have been preliminarily employed for the delivery of active molecules or targeted proteins *in vivo* (Zhou et al., 2018),However, delivering BMP-2 in a low-dose yet effective manner remains a significant challenge (Issa et al., 2008). Research by Zhou et al. has demonstrated that 2-N, 6-O-sulfated chitosan (26SCS) can synergistically enhance the biological activity of BMP-2 at lower doses (Zhou et al., 2009). By binding with BMP-2, 26SCS significantly boosts the bioactivity of BMP-2 under low-dose conditions. Nonetheless, leveraging relatively low doses of BMP-2 to repair critical-sized bone defects and reconstruct the microvascular network remains a considerable challenge (Cao et al., 2014).

Moreover, the duration of BMP-2 release has a significant impact on bone regeneration. Research by MayLin T. Howard et al. indicates that sustained release of BMP-2 offers a markedly improved bone regeneration effect compared to short-term release (Howard et al., 2022). To extend the release duration of BMP-2, researchers have developed polydopamine-coated PLLA nanofibers and PLLA-carbon nanotube-microhydroxyapatite composite scaffolds (Cho et al., 2014; van der Zande et al., 2011). The former can maintain protein release for up to 28 days and induce substantial bone regeneration at the site of fibular defects in mice (Cho et al., 2014). Thus, an ideal BMP-2-containing bioactive scaffold for local bone regeneration should enable precise control and sustained release of BMP-2, thereby minimising the risk of ectopic bone formation. Eun Young Jeon et al. designed a fucoidan-PLL complex coacervate, which effectively encapsulates BMP-2 in a simple and rapid manner, achieving an encapsulation efficiency of approximately 98.7% (Jeon et al., 2022). Furthermore, BMP-2 is released continuously and precisely at the target site. *In vitro* and *in vivo* experiments have validated its excellent bioactivity and local bone regeneration capability.

The process of osteogenesis involves a series of cellular events, including proliferation, osteogenic commitment, maturation, and matrix mineralization, all of which are tightly regulated by several key biomacromolecules (James et al., 2016; Huang et al., 2007; Wu et al., 2016). BMP-2 activates the Smad-dependent osteogenic signaling pathway by binding to the heteromeric receptors BMPR1 and BMPR2, which subsequently triggers the phosphorylation of R-Smad proteins (Huang et al., 2007; Gu et al., 2017; Wan et al., 2006). The activated R-Smad proteins bind to CoSmad to form a complex that further regulates the expression of osteogenic transcription factors such as Runx2 and OSX (Huang et al., 2007). It has been reportedthat upregulation of BMPR1 and BMPR2 expression enhances the osteogenic potential of stem cells, whereas their expression is significantly reduced during adipogenesis (Wan et al., 2006).

This systematic review also has several limitations. Firstly, many of the included studies did not report key methodologies to avoid bias, such as blinding, and nearly half of the studies did not clearly state whether randomisation was performed. For instance, some studies neither reported the randomisation process nor clarified whether outcome assessments were conducted in a blinded manner, which may increase the risk of misunderstanding the impact of BMP-2 loaded 3D printed scaffolds on bone regeneration. Secondly, the included studies exhibited significant variability in terms of animal species, bone defect size, treatment duration, materials used for 3D printed scaffolds, and dosages of BMP-2, resulting in considerable statistical heterogeneity in this meta-analysis. To mitigate this issue, we employed a random-effects model and conducted sub-group analyses based on factors such as animal species, bone defect size, and treatment duration; however, some results still failed to effectively reduce heterogeneity. Furthermore, due to the limited data available from the included studies, we were unable to conduct a subgroup analysis for new bone area, indicating the need for more comprehensive research to validate these findings. Thirdly, there was considerable variability in the materials used for 3D printed scaffolds (including porosity and pore size) and the methods of BMP-2 loading, with the majority of the literature failing to report specific BMP-2 loading doses. This lack of detailed information limits further subgroup analyses. Fourthly, due to the limited number of included studies, the results may be subject to certain biases. For instance, in the assessment of new bone volume, studies with treatment durations exceeding 8 weeks reported lower effect sizes, whereas those with treatment durations of 8 weeks or less exhibited higher effect sizes. Therefore, the results of this review should be interpreted with caution. Future studies should be conducted in a more standardized manner to further validate the conclusions.

Currently, there are few reports on the clinical trials of BMP-2-loaded 3D-printed scaffolds. However, several issues need to be thoroughly investigated before they can be applied clinically. Firstly, the selection of materials for 3D-printed scaffolds is crucial. The materials involved in the studies included in this review comprise PCL, PLA, and sodium alginate, among others, all of which are widely used in the fabrication of scaffolds for bone regeneration. An ideal 3D-printed scaffold should possess biodegradability, but after loading with BMP-2, its degradation rate must be appropriately maintained to ensure a sustained release of BMP-2 to effectively promote bone regeneration. B.B. Seo et al. demonstrated that by loading BMP-2 into a polyphosphonyl phenyl hydrogel system, complete degradation of the hydrogel was achieved within 21 days, accompanied by a sustained release of BMP-2, thereby promoting bone regeneration (Seo et al., 2015). Additionally, there is a lack of an objective standard for the dosage of BMP-2. In this review, the concentrations of BMP-2 loaded into the scaffolds across various studies ranged from 0.3 µg to 100 μg, with ten publications not specifying the exact dosage. While some studies have reported BMP-2 concentrations, they did not investigate the effects of different dosages on bone regeneration. Determining the optimal dosage of BMP-2 during scaffold implantation remains a significant issue, as varying volumes of bone defects may require different dosages to ensure effective bone induction while minimizing the risk of complications. Lastly, the majority of studies have been conducted using experimental bone defect models in healthy animals. However, bone defects in pathological tissues are often associated with underlying diseases, and their healing responses may differ from those observed in healthy animal models. In fact, the tissue environments created by the pathogenic mechanisms underlying bone defects differ significantly from those generated in experimentally induced bone defects. This distinction is particularly important to consider in clinical applications.

In conclusion, there is a pressing need for further research on 3D printed scaffolds loaded with BMP-2 to promote bone regeneration. Attention should not only be given to the specific research methodologies for these scaffolds and the optimal dosage of BMP-2, but also to the exploration of how to translate findings from animal models to larger animal species, as large animals share more anatomical and physiological similarities with humans (Walters et al., 2017).

## 5 Conclusion

This meta-analysis confirms that BMP-2-loaded 3D-printed scaffolds significantly improve key bone regeneration indicators, such as BV/TV, new bone volume, and percentage of new bone area. 3D printing technology not only provides ideal mechanical support for the scaffolds but also, in synergy with BMP-2, creates a favourable microenvironment for bone regeneration through mechanisms such as inducing osteoblast chemotaxis, regulating osteogenic factor expression, and promoting angiogenesis. Based on the current evidence, future research should focus on: 1) long-term safety and efficacy evaluations in large animal models; 2) optimisation of BMP-2 dosage and scaffold parameters in preclinical trials; and 3) establishment of standardised evaluation systems. Advancing these research directions will accelerate the translation of this technology from laboratory studies to clinical applications, providing new treatment options for patients with bone defects. The results of this study offer valuable guidance for future research in this field.

## Data Availability

The original contributions presented in the study are included in the article/Supplementary Material, further inquiries can be directed to the corresponding authors.

## Abbreviations

3D, Three-dimensional; BMP-2, Bone Morphogenetic Protein-2; BV/TV, Bone Volume to Total Volume ratio; SMD, Standardised Mean Difference; CIs, Confidence Intervals.
