# Supplementary material for: 3D printed scaffolds loaded with BMP-2 for bone defect regeneration: a systematic review and meta-analysis
Source: Front Physiol. 2025 Jul 30;16:1641937. doi: 10.3389/fphys.2025.1641937 (PMC12343735; doi:10.3389/fphys.2025.1641937)
Supplement: Supplementary file 1 [file Table1.docx]

**Supplementary Table_1** Eegg's test and Bgger's test results for different outcome metrics

|  | Eegg's test | Bgger's test |
| --- | --- | --- |
| BV/TV | P=0.037 | P=0.029 |
| New bone volume | P=0.000 | P=0.000 |
| New bone areas | P=0.056 | P=0.133 |
