# Supplementary figures and images for: 3D printed scaffolds loaded with BMP-2 for bone defect regeneration: a systematic review and meta-analysis
Source: Front Physiol. 2025 Jul 30;16:1641937. doi: 10.3389/fphys.2025.1641937 (PMC12343735; doi:10.3389/fphys.2025.1641937)

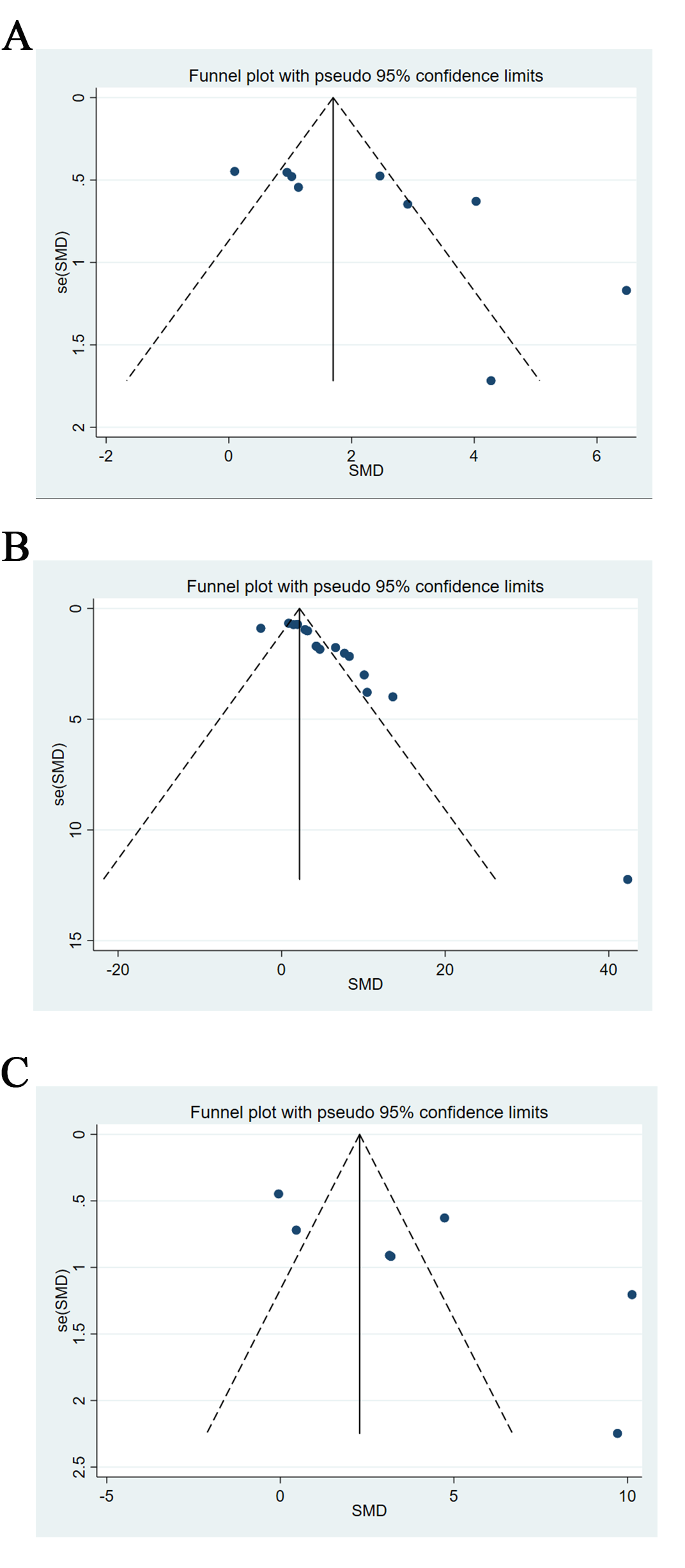

Supplement: Supplementary file 2 [file Image1.tif]
